# Discordance between self-report and clinical diagnosis of Internet gaming disorder in adolescents

**DOI:** 10.1038/s41598-018-28478-8

**Published:** 2018-07-04

**Authors:** Hyunsuk Jeong, Hyeon Woo Yim, Seung-Yup Lee, Hae Kook Lee, Marc N. Potenza, Jung-Hye Kwon, Hoon Jung Koo, Yong-Sil Kweon, Soo-young Bhang, Jung-Seok Choi

**Affiliations:** 1 Department of Preventive Medicine, College of Medicine, The Catholic University of Korea, Seoul, Korea; 2 Department of Psychiatry, College of Medicine, The Catholic University of Korea, Seoul, Korea; 3 Department of Psychiatry, Yale University, Connecticut, USA; 4 Department of Psychology, Korea University, Seoul, Korea; 5 Department of Psychology and Child, College of Human Services, Hanshin University, Osan, Korea; 6 Department of Psychiatry, Eulji University Eulji General Hospital, Seoul, Korea; 7 Department of Psychiatry, SMG-SNU Boramae Medical Center, Seoul, Korea

## Abstract

This study aimed to estimate overreporting (the false positive) and underreporting (false negative) rates in self-reported IGD assessment compared with clinical diagnosed IGD. The study population consisted of 45 with IGD and 228 without IGD based on clinical diagnosis from the Internet User Cohort for Unbiased Recognition of Gaming Disorder in Early Adolescence (iCURE) study. All participants completed self-reported IGD assessments. Clinical interviews were conducted blindly by trained mental health professionals based on DSM-5 IGD criteria. Self-assessed average daily amount of gaming time and game genre were measured. Psychological characteristics, including anxiety, suicidality, aggression, self-control, self-esteem, and family support, were obtained from the baseline survey. The false-negative rate for self-reported IGD assessment was 44%. The false-negative group reported less time playing online games than the IGD group, though their psychological characteristics were similar to those of the IGD group. The false-positive rate was 9.6%. They reported more time playing online games than non-IGD group, though their psychological characteristics were similar to those of non-IGD group except self-control. The discrepancy of IGD diagnoses between self-reports and clinical diagnosis revealed limitations of self-measurements. Various strategies are required to overcome the methodological shortfalls of self-reports for the assessment of IGD.

## Introduction

Self-report measures are an inexpensive and relatively quick way to collect much data. However, these measures may lack credibility due to biased responding. In general, research participants may respond in a way that makes them look as good as possible. In particular, self-report biases may be introduced when responding to sensitive questions, such as those related with addictive behaviours, sexual experiences, and delinquency. Self- measurements may also be prone to exaggeration. Some evidence suggests that individuals tend to over-report their pro-environmental behaviour^1^.

In 2013, the Diagnostic and Statistical Manual of Mental Disorders, Fifth Edition (DSM-5) proposed diagnostic criteria for Internet gaming disorder (IGD) as a condition warranting further empirical and clinical research. The suggested IGD criteria paved the way for measuring IGD in a consistent manner. Many studies have used the DSM-5 diagnostic criteria to estimate the prevalence of IGD, and most have confirmed IGD using self-administered questionnaires^2–7^. Several IGD assessments have been examined to test the diagnostic validity of DSM-5 IGD criteria including the 10-item Internet Gaming Disorder Test (IGDT-10)^8^, IGD-20^9^, IGDS-SF9^10^, Video Game Dependency Scale (CASA)^6^, and IGD scales with both polychotomous and dichotomous methods to determine the most practical scale for IGD diagnosis^11^. Population based surveys did not include clinician diagnosis but used Latent Class Analysis in the group with the highest likelihood of meeting the nine criteria as the gold standard to investigate the cut-off threshold proposed in the DSM-5^8,9^.

Although self-reports may have limitations in psychiatric epidemiological studies, few studies have considered the accuracy of self-reported assessment in making a diagnosis of IGD. A previous study reported that the sensitivity and specificity of self-reported Internet addiction measurement were around 85%, indicating good diagnostic accuracy^12^. However, false positive and false negative results were still present in the self-report assessment. To the best of our knowledge, no studies have been conducted comparing self-reported IGD with clinical interviews based on DSM-5 criteria in a population-based study. Therefore, this study aimed to estimate the false-positive and false-negative rates in an IGD self-report assessment compared with a clinical assessment of IGD made by mental health professionals based on DSM-5 IGD diagnostic criteria. The false-negative (underreporting) group was defined as adolescents who did not have IGD on self-report, but were diagnosed with IGD in the clinical diagnostic interview. The false-positive (overreporting) group was defined as adolescents who had IGD on self-report, but who were assessed as not having IGD in the clinical diagnostic interview.

Because prevalence estimates using self-reported assessment can be biased because of false-negative and false positive rates, it is important to estimate the magnitudes and direction of bias by measuring false-negative and false positive rates. Longitudinal follow-up studies will reveal whether the discrepancies between self-reported assessments and clinical interview were genuine IGD or not. However, since the current study was based on the baseline results, we performed an exploratory analysis to evaluate whether the false-negative and the false positive groups were related to the non-IGD or IGD group. IGD risk factors can predict who is more likely to become addicted^13^. This study investigated whether psychosocial characteristics which are well known risk and protective factors related to the development of IGD such as anxiety^14^, suicidality^15^, aggression^16^, self-control^17^, self-esteem^18^, and family support^19,20^ differed between adolescents in the false-positive and false-negative groups. We hypothesized that the false positive group was related to the non-IGD group, and the false negative group was related to the IGD group with regard to psychological characteristics related to development of IGD.

## Results

Table 1 compares the socio-demographic characteristics of the 45 participants with IGD and the 228 without IGD from the 273 case-sub-cohort participants. The gender distribution did not differ between the two groups. There was a modest difference in family type: the frequency of non-intact families was 22.2% in the IGD and 11.4% in the non-IGD groups (*P* = 0.050).

**Table 1:** Socio-demographic characteristics of 273 adolescents with or without IGD.

Variables	IGD (n = 45)	Non-IGD (n = 228)	*P-value*
Gender			0.812
Male	24 (53.3)	126 (55.3)	
Female	21 (46.7)	102 (44.7)	
Family type			0.050
Intact	35 (77.8)	202 (88.6)	
Broken	10 (22.2)	26 (11.4)	
Time when internet gaming began			<0.001
Kindergarten	18 (40.0)	28 (12.3)	
1–3 grades	15 (33.3)	96 (42.3)	
≥4 grade	11 (24.4)	67 (29.5)	
Never	1 (2.2)	36 (15.4)	

In the IGD group, 18 (40.0%) had started playing Internet games before entering elementary school, 15 (33.3%) started between the first and third grades, and 11 (24.4%) started in or after the fourth grade. In the non-IGD group, 28 (12.3%) had started playing Internet games before entering elementary school, 96 (42.3%) started between the first and third grades, and 67 (29.5%) started in or after the fourth grade. The IGD group began playing Internet games earlier than the non-IGD group (*P* < 0.001).

The self-reported IGD assessment had a sensitivity of 55.6% (95% CI: 45.2–67.1%) and specificity of 90.4% (95% CI: 83.0–97.2%) (Table 2). Considering clinical diagnosis as a gold standard, a sensitivity of 55.6% meant that a self-reported IGD measurement was detected in 55.6% of the sample population, while 44.4% of cases were undetected. A specificity of 90.4% meant that self-reported IGD assessment of non-IGD adolescents was correctly identified. The false-positive rate was 9.6% (95% CI: 6.4–14.2%), and the false-negative rate was 44% (95% CI: 30.9–58.8%).

**Table 2:** Prevalence and concordance of IGD diagnosis between self-report and clinical diagnosis among seventh-grade students.

		Clinical diagnosis	
		IGD	Non-IGD
Self-report assessment	Positive IGD	25	22
	Negative IGD	20	206
		45 Sensitivity: 56.6% (95% CI: 45.2–67.1%)	228 Specificity: 90.4% (95% CI: 83.0–97.1%)

The four groups did not differ in the type of games they reported playing. For the self-reported average daily amount of time spent playing online games during weekdays and weekend days, the false-negative adolescents reported spending less time playing online games than the IGD adolescents. The false-positive group reported more time playing online games than non-IGD adolescents. These differences were statistically significant (*P* < 0.001) (Table 3).

**Table 3:** Gaming behaviours of the four groups based on information from the self-report and clinical interview.

Variables	Concordance^†^ (n = 231, 84.6%)		Discordance^‡^ (n = 42, 12.4%)		*P*-value
	Non-IGD^§^ (n = 206)	IGD^∥^ (n = 25)	False positive^¶^ (n = 22)	False negative^#^ (n = 20)	
Current online game player	80 (38.8)	13 (52.0)	11 (50.0)	9 (45.0)	0.476
Type of game played					
Role playing	53 (25.7)	10 (40.0)	7 (31.8)	4 (20.0)	0.383
Shooter	48 (23.3)	10 (40.0)	8 (36.4)	3 (15.0)	0.119
Simulation	44 (21.4)	8 (32.0)	8 (36.4)	5 (25.0)	0.317
Sports	28 (13.6)	2 (8.0)	6 (27.3)	1 (5.0)	0.142
Arcade	82 (39.8)	11 (44.0)	7 (31.8)	7 (35.0)	0.819
Time online gaming during weekdays (min/day)	65.1 ± 85.5	215.2 ± 156.2	132.7 ± 81.3	96.3 ± 125.9	<0.001
Time online gaming on the weekend (min/day)	116.6 ± 148.4	365.6 ± 228.9	212.3 ± 132.9	160.3 ± 208.3	<0.001

^†^Concordance: the same results were obtained by self-report and clinical diagnosis.

^‡^Discordance: different results were obtained by self-report and clinical diagnosis.

^§^Non-IGD: neither self-report nor clinical diagnosis of IGD.

^∥^IGD: both self-report and clinical diagnosis of IGD.

^¶^False positive: participants who were classified as IGD suspects by self-reports, but who were confirmed negative by diagnostic interview.

^#^False-negative: defined as people who were reported as normal by self-report but who were diagnosed with IGD by mental health specialists.

All psychological characteristics were significantly different among the four groups in ANOVA and Chi-square test. Anxiety, aggression, self-esteem, familial support, and experience of suicidality during the past year were not different between non-IGD and false positive groups in Duncan and Dunn post-hoc analyses. The false negative group was similar to the IGD group with regard to the five psychosocial characteristics. Compared to non-IGD, the mean scores of self-control were high in the false positive and false negative groups (Table 4).

**Table 4:** Intergroup differences on psychological characteristics.

Variables	Non-IGD (n = 206)	IGD (n = 25)	False positive (n = 22)	False negative (n = 20)	P-Value
Anxiety	29.8 ± 7.7^C^	39.8 ± 10.7^A^	33.1 ± 7.7^BC^	35.8 ± 10.0^AB^	<0.001^*†^
Aggression	56.8 ± 16.4^B^	72.6 ± 23.7^A^	64.3 ± 15.^AB^	70.3 ± 15.3^A^	<0.001^*†^
Self-control	16.6 ± 4.3^B^	21.1 ± 6.5^A^	19.3 ± 4.8^A^	20.2 ± 5.6^A^	<0.001^*†^
Self-esteem	31.0 ± 5.8^A^	24.8 ± 7.1^C^	28.9 ± 4.9^AB^	26.3 ± 5.0^BC^	<0.001^*†^
Familial support	34.4 ± 6.3^A^	25.7 ± 8.6^C^	31.8 ± 7.6^AB^	30.4 ± 6.5^B^	<0.001^*†^
Suicidality	28 (13.6)^B^	8 (32.0)^A^	4 (18.2)^B^	7 (35.0)^A^	0.017^‡§^

Numbers are presented as mean ± standard deviation or n (%).

^*^P values were calculated by ANOVA test.

^†^Post hoc analysis used the Duncan method.

^‡^P value was calculated by Chi-square test.

^§^Post hoc analysis used the Dunn method.

Means with the same alphabetical capital letter are not significantly different.

## Discussion

This study identified some discrepancies between self-measurements and clinically verified IGD diagnoses among adolescents. The false-negative rate was 44% (95% CI: 31.9–58.8%), and the false-positive rate was 9.6% (95% CI: 6.4–14.2%). Several factors may explain the false-negative findings, including social desirability effects, a dislike for participating in the study, and limitations of self-assessment tools.

It can be difficult to accurately assess risk behaviours in adolescents with self-reported questionnaires^21^. Adolescents at risk for various disorders may not wish to disclose certain facts about themselves, and they may purposefully under-report socially undesirable risk behaviours. Social desirability effects can threaten validity of results because responses are, by definition, systematically biased. Respondents, wishing to present themselves in a favorable light, may consciously or unconsciously tailor their answers to make their behaviours appear less deviant and more socially desirable^22,23^. Williams *et al*.^23^ postulated that social-desirability bias leads players to underreport their playing time^23^. Individuals playing online games may also under-report gaming time for other reasons (e.g., losing track of time while immersed in the activity). The mode of data collection may influence the degree of social-desirability bias^22,24^. Research on this topic has found that the further a mode is from face-to-face interaction, the less is the extent of social-desirability bias. It should also be noted that some respondents may over-rate participation in socially deviant behaviours, particularly during developmental stages like adolescence that are characterized by an increase in seemingly rebellious behaviours.

Some adolescents simply might not have been inclined to participate in the survey itself. Perhaps they considered the survey tedious and uninteresting; as a result, it is not possible to rule out reluctant answering. Unwillingness to participate could have affected the false negative rate when considering different situations between self-report and clinical interview. Self-report assessment administered by unit of class, 30 adolescents was connected the website in the computer room at the same time without any enforcement, but clinical interview was conducted through 1:1 interview by psychiatrists and certified clinical psychologists. In the context of 1:1 interviews with a probing process to identify IGD symptoms, students would have been less affected by their willingness to participate than in a self-administered survey.

The discrepancy between self-reported and clinically diagnosed IGD might also be due to the limitations of the “yes” or “no” response method as a self-reported assessment tool. When IGD was assessed using self-report among adolescents with the dichotomous response method of “yes” or “no,” the respondents might have faced difficulties accurately describing their responses in nuanced situations. This may have introduced error because the response type forced the responder to make a choice between the two options. The inherent limitations of a “yes” or “no” response-based questionnaire make it challenging to obtain precise responses from subjects in assessing IGD based on DSM-5. To overcome the limitations of self-report assessment tools, clinicians or mental health specialists may need to evaluate IGD symptoms through a probing process.

The false-negative adolescents reported less time playing online games than IGD adolescents. However, the assessments of psychological characteristics showed similar results between the false-negative and IGD groups of adolescents in all assessments. The false-negative group might have under-reported their game-use behaviour or IGD symptoms in self-report assessments. Twenty of 45 IGD case produced a false negative response. Among these 20, four adolescents responded insincerely by marking all of the same numbers on the first stage self-report IGD assessment (IGUESS). They even responded with the same number on the reverse questions, contradictory to logic, which were included to assess insincere responses in the self-report assessment. Another four adolescents appeared to carefully review the entire questionnaire and reported no IGD symptoms. Twelve of 20 false negative adolescents might have underreported their IGD symptoms due to social desirability or the results of the limitations of self-assessment tools.

In Korea, the government-led youth game use factual survey is evaluated every year for 1^st^-, 4^th^-, 7^th^-, and 9^th^-grade students. Once students are classified into the high-risk group, they will be guided to receive an intervention. This process may make the false-negative rate of Korea higher than other countries.

The false-positive group reported more time playing online games than non-IGD adolescents. However, they showed a similar pattern to the non-IGD group of adolescents in most psychological characteristics, with the exception of self-control. Lack of self-control might be increased impulsiveness. Externalizing characteristics may be related to the behaviours of exaggeration or boasting about game skills or about the perception of power or influence in a peer group of adolescents.

The prevalence of IGD was 1.94% (45 of 2,319), which may be an underestimate. To determine the overall prevalence of IGD, we randomly sampled 232 adolescents out of the 2,319 (10%) enrolled in the iCURE study. Among 232, 73 were met the inclusion criteria for the second-stage clinical diagnostic interview, and 153 did not meet the criteria. Among 153, two were diagnosed with IGD in the clinical interview, although they did not meet the inclusion criteria for the second-stage clinical diagnostic interview. Based on these results, 20 adolescents were likely to be diagnosed with IGD among the adolescents who did not meet the criteria for the second-stage clinical diagnostic interview. However, we identified only 8 IGD cases through the second-stage diagnostic interview criteria, so we estimate that approximately 12 IGD cases may have been missed.

This study had several limitations. First, we selected a representative sample of 10% from the total population due to practical concerns, but only four of these individuals were diagnosed with IGD (two false-negative, two true-positive). Because the representative sample size was relatively small, our results may have been affected by random variability.

Second, clinically diagnosed IGD was evaluated by only one mental health professional per each person rather than by multiple observers. However, the diagnostic evaluations occurred through four-day workshops to ensure the reliability of the diagnosis. Although the mental health specialists conducted clinical diagnostic interviews according to the DSM-5 diagnostic criteria, it may have been difficult to determine whether the self-report was false-positive or false-negative because the threshold of the DSM-5 IGD diagnostic criteria have not been clearly established through formal testing. Considering an IGUESS cutoff score for IGD risk of 10 or higher, and an IGUESS score of 6 points is expected to be associated with about 2 subclinical symptoms, almost all of the adolescents with problems in gaming behaviours from the entire cohort were likely to have undergone the second-stage clinical diagnostic interview, so we believe that most cases were confirmed. We also included in the second-stage diagnostic interview anyone who responded insincerely by marking all of the same numbers on the IGUESS or anyone who reported a suicidal experience during the past year. These efforts helped ensure that we could identify all participants with IGD.

Despite these efforts, we cannot exclude the possibility that the prospective second-stage diagnostic interview missed participants with IGD because of the limitations of self-report. Two of four adolescents were diagnosed with IGD among the random sample; it is possible that the false-negative rate was an underestimate. Self-reported assessment is a convenient survey method, but it cannot always detect false reporting by respondents, so the results might be biased. The reason for the high false-negative rate may be due to repetitive exposure to similar questions among Korean students.

Self-reported IGD assessments have the possibility of introducing bias which would affect the validity of the measurement. To overcome the methodological shortfalls of self-reports for the assessment of IGD, future research may incorporate other objective measurements, include reverse questionnaires with contradictory to logic, use Likert scale assessment, or apply randomized response techniques for gathering self-reported assessment in sensitive questions with protection of privacy^25^.

Adolescents in the false positive or false negative group could be in a subclinical stage. Natural history of false positive and false negative groups should be examined to determine whether they belong to the IGD or non-IGD group through longitudinal follow-up. Since the iCURE study was designed as a prospective longitudinal study, the results will be revealed in future studies.

Because the false-positive or false-negative rate may be influenced by various personal, environmental, or situational factors, it is difficult to generalize our results to other age groups, populations of different ethnic, racial or cultural compositions, or individuals living in rural areas or other countries. In addition, although the sub-cohort participants consisted of a representative sample from 21 schools, most schools included in the iCURE study were registered in Seoul, so the results may be most relevant to a metropolitan environment.

## Methods

### Participants and study design

In 2015 through 2017, baseline assessment for the **I**nternet User **C**ohort for **U**nbiased **R**ecognition of Gaming Disorder in **E**arly Adolescence (iCURE) study was conducted in Korea. The study was fully reviewed and approved by the Institutional Review Board of The Catholic University of Korea (MC140NM10085) and conducted in accordance with the Declaration of Helsinki in 2013^26^. Informed consent was acquired from all participants and their parents or legal guardians following explanation of the nature of the principles of research, including confidentiality and the freedom of choice to participate. In addition, the current study received approval from the Institutional Review Board of The Catholic University of Korea for data analysis (MC17ENSI0108). The iCURE data management board released de-identified iCURE data.

The protocol for the iCURE study has been published previously^27^. The iCURE study has enrolled 2,319 adolescents from the 3^rd,^ 4^th^ and 7^th^ grades. All participants completed self-administered IGD assessments using both the Internet Game Use-Elicited Symptom Screen (IGUESS)^28^ and the Internet Gaming Disorder Questionnaire (IGDQ) as a self-report version of the highly Structured Clinical Interview for DSM-5 IGD (SCI-IGD)^29^. A second-stage assessment for making a clinical diagnosis was made for those participants who had one of the following indications: a score of six or higher on the IGUESS; anyone who responded insincerely by marking all of the same numbers on the IGUESS including the reverse questions in contradictory to logic; or anyone who reported a suicidal experience during the past year. These participants were given diagnostic interviews. Participants who scored 6 points in the IGUESS were expected to have about two subclinical IGD symptoms. A total of 834 among 2,319 adolescents participated in the second-stage clinical diagnostic interview. Forty-three adolescents were verified as having IGD through the two-phase assessment.

**Figure 1:**
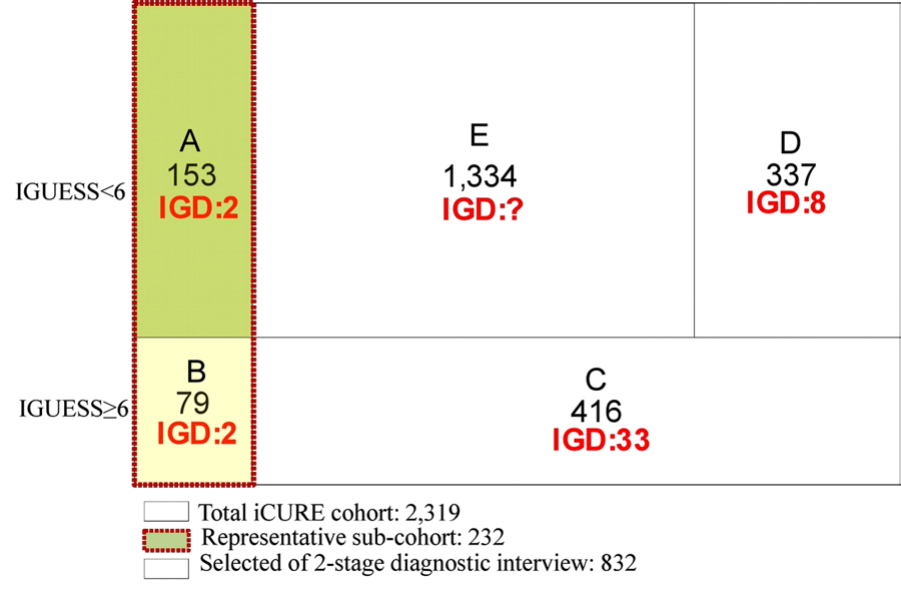
Study population and participants included in the data analysis. (**A**,**B**) Representative sub-cohort; (**A**) Representative sub-cohort and IGUESS < 6. (**B**) Representative sub-cohort and IGUESS ≥ 6. (**B**–**D**) Selected for the 2-stage diagnostic interview (2-SDI). (**C**) 2-SDI because of IGUESS ≥ 6. (**D**) 2-SDI despite of IGUESS < 6 (e.g. insincere response, reported suicidality during the past-year).

Two hundred thirty-two adolescents were selected as a representative random sample of 10% of the iCURE study participants, stratified by school. All of these adolescents completed the clinical diagnostic interviews. Some of these adolescents were part of both the representative sample and the second-stage diagnostic interviews. Of the 232 adolescents, 4 were diagnosed with IGD in the clinical interviews, including 2 who were included in the second-stage clinical diagnostic process and 2 who did not meet the second-stage clinical-interview indications but were diagnosed with IGD through the clinical diagnostic interview. This case sub-cohort consisted of 45 participants with IGD (case) and 228 participants without IGD (non-case), as diagnosed clinically (Fig. 1).

### Measurements

#### Self-reported IGD assessment

The IGDQ^29^ was used for self-reported IGD assessment. This assessment included “yes” or “no” responses on web-based surveys. Participants who responded “yes” to five or more of the nine IGD criteria were classified as having “self-reported IGD.” The original version of the IGDQ had good reliability (Cronbach’s α = 0.85) and structural validity (TLI = 0.95, CFI = 0.96 and RMSEA = 0.06). Moderate to strong correlations with depressive symptoms, anxiety, hyperactivity, and gaming hours supported the criterion validity of this scale (in submission). Meeting five or more of the DSM-5 criteria was revealed to be the best cut-off to distinguish the normal group from the clinically impaired group^29^.

#### Clinically diagnosed IGD

The clinical diagnostic interviews for IGD were conducted face-to-face by five psychiatrists and four experienced clinical psychologists. To ensure reliability, all of the mental health specialists who participated in the clinical interviews completed a four-day workshop. They were kept blind to the results of the self-reported assessments. At the end of each day of diagnostic interviews, they discussed any ambiguous diagnoses and to make final determinations. The interviews assessed participants based on the nine DSM-5 IGD criteria, in accordance with the international consensus for IGD represented in the DSM-5. Individuals with positive responses to five or more of the IGD criteria and functional impairments were classified into the IGD group. The interviews were confidentially conducted in an independent space within the participants’ schools. The overall IGD diagnostic percent agreement was 91%, and overall percent agreement in each of the nine IGD criteria ranged from 79% to 90%.

#### Socio-demographic factors

Socio-demographic characteristics, including gender and family structure (e.g., intact or separated family structures in the primary residence of the participant), were obtained from the iCURE study baseline assessment data.

#### Online-gaming-related questions

Self-reported assessments of participants’ gaming usage were obtained from the baseline survey, including questions regarding first exposure, average daily amount of time spent playing online games during weekdays and weekend days, and the titles of the games that they had played during the past 12 months. Online games were classified into 5 categories: role-playing games, shooter games, simulation games, arcade games, and unknown.

#### Psychological characteristics

Psychological characteristics of anxiety, suicidality, aggression, self-control, self-esteem, and family support were obtained to evaluate the psychological profiles of the self-assessed IGD and clinically diagnosed IGD groups compared with those of adolescents without positive assessments of IGD.

To examine trait anxiety, we used the Korean translation of the Trait Anxiety Inventory for Children (TAIC) developed by Spielberger and colleagues. The Korean TAIC is a 3-point 20-item inventory that asks respondents to indicate how frequently they feel worried, bothered, or nervous. The total score ranges from 20 to 60^30^. This scale is reliable and showed a Cronbach’s alpha of 0.91 in the current study.

The presence of suicidal ideation, suicide plans, and suicide attempts was determined through the following direct questions derived from the Structured Clinical Interview for DSM-IV Axis I Disorders (SCID-I)^31^. Participants were asked whether they had seriously considered committing suicide, made a plan to commit suicide, or had attempted suicide during the past year. The presence or absence of suicide ideation, plans, and attempts was based on the participant response (yes or no).

Self-esteem was measured using 10 items from the Self-Esteem Scale^32^. This measure detects feelings of self-acceptance, self-respect, and generally positive self-evaluation. Each item is rated on a 5-point Likert scale, ranging from 1 (strongly disagree) to 5 (strongly agree). The total score ranges from 10 to 50. This measure detects feelings of self-acceptance, self-respect, and generally positive self-evaluation. In the current study, this scale was reliable, with a Cronbach’s alpha of 0.83.

Self-control was assessed with the 10-item Self-Control Scale of Gottfredson and Hirschi (1990)^33^. Each item is rated on a 4-point scale: (4) strongly agree, (3) somewhat agree, (2) somewhat disagree, and (1) strongly disagree. Higher scores indicate greater problem with self-control. This scale was reliable, with a Cronbach’s alpha of 0.86 in the current study.

Aggression was evaluated by the Buss-Perry Aggression Questionnaire with a 29-item, 5-point Likert scale measurement. Higher scores indicate greater aggression^34^. This scale was reliable, with a Cronbach’s alpha of 0.88 in the current study.

Social support was evaluated with the Social Support Appraisals Scale (SSAS). This scale evaluated the child’s subjective appraisal of family, peer, and teacher support to address perceived support. It is a 41-item self-report instrument developed by Dubow *et al*.^35^. Items are rated on a 5-point Likert scale, from 1 (never) to 5 (always). Peer support was used in the analysis. Higher scores indicated that the participant perceived greater social support from peers^35^. This scale was reliable, with a Cronbach’s alpha of 0.94.

### Statistical analyses

Socio-demographic characteristics were summarized with numbers and percentages for categorical variables or means and standard deviations (SDs) for continuous variables. The study population was divided into four groups according to either concordance or discordance of the self-reported and clinically diagnosed IGD. The non-IGD comparison group included participants who were evaluated as not having IGD in both the self-report and clinical interview. The IGD group included anyone who had IGD on both the self-report and clinical interview. The false-negative group included adolescents who did not have IGD on self-report, but were diagnosed with IGD in the clinical diagnostic interview. The false-positive group included adolescents who had IGD on self-report, but who were assessed as not having IGD in the clinical diagnostic interview. False positive rate is calculated as the fraction of the number of classified as non-IGD in clinical diagnosis as the denominator and the number evaluated an IGD in self-reported assessment as the numerator. False negative rate is calculated as the fraction of the number of patients classified as IGD in clinical diagnosis as the denominator and the number of patients evaluated as non-IGD in self-reported assessment as the numerator. Sensitivity and specificity were calculated, considering the mental health professionals’ clinical interviews as the gold standard.

The t-test, chi-square test, and Fisher exact test were used to determine differences between the four groups.

Differences in psychological characteristics among the four groups were analysed using ANOVA for continuous variables and Chi-square tests for categorical variables. If there was a significant group difference in ANOVA, Duncan multiple comparison test was used for post-hoc comparison. If there was a significant group difference in Chi-square test, Dunn multiple comparison test was used for post-hoc comparison. All tests are two-tailed, and p-values less than 0.05 were considered statistically significant. All analyses were performed with SAS software package version 9.4 (SAS Institute Inc., Cary, NC, USA).

### Data Availability

The datasets generated during and/or analysed during the current study are available from the corresponding author.
